# Managing a Right Coronary Artery Perforation, Pseudoaneurysm, and the Fight for Patency

**DOI:** 10.1016/j.jaccas.2024.103092

**Published:** 2025-02-05

**Authors:** Maruf Sarwar, Stephen D. Adedokun, Keonmin Hwang, Mahesh Anantha Narayanan

**Affiliations:** a Section of Internal Medicine, White River Health, Batesville, Arkansas, USA; b Section of Cardiovascular Disease, University of Tennessee Health and Science, Memphis, Tennessee, USA; c Thomas F. Frist, Jr. College of Medicine at Belmont University, Nashville, Tennessee, USA; d Section of Cardiovascular Diseases, White River Health, Batesville, Arkansas, USA; e University of Arkansas Medical Sciences, Little Rock, Arkansas, USA

**Keywords:** acute coronary syndrome (ACS), percutaneous coronary intervention (PCI), coronary perforation, ping-pong technique, right coronary artery, Ellis type III perforation, Balloon tamponade, pseudoaneurysm, coil embolization, interventional cardiology, dual-catheter approach, myocardial infarction (MI), stent thrombosis, pericardiocentesis, coronary, angiography

## Abstract

Managing coronary perforation under anticoagulation presents a clinical dilemma. This case explores the challenge of preserving vessel patency while addressing perforation. A 76-year-old woman presented with acute chest pain and inferior ST-segment depression. Angiography revealed a small-caliber right coronary artery with faint collateral flow. During percutaneous coronary intervention, an Ellis type III perforation occurred, managed with balloon tamponade and pericardiocentesis. Using the ping-pong technique, flow was restored without reversing anticoagulation. A pseudoaneurysm developed at the perforation site, which was treated successfully with coil embolization. This case highlights the balance required between bleeding risk and ischemic burden in acute coronary syndrome. It underscores the importance of advanced percutaneous coronary intervention techniques like the ping-pong strategy for effective intervention and management of complications such as pseudoaneurysms.

## Visual Summary

**Figure:**
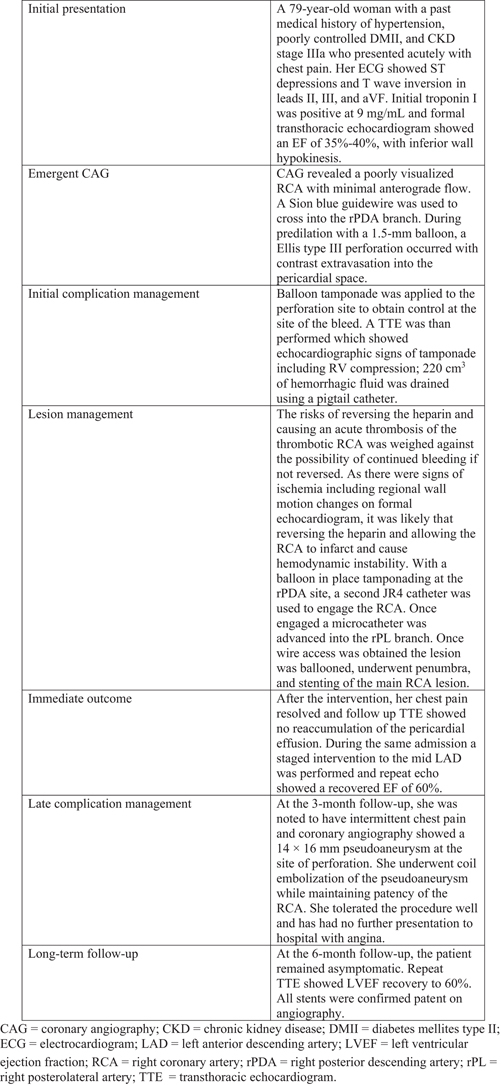

**Visual Summary:** Timeline of Events

Initial presentation	A 79-year-old woman with a past medical history of hypertension, poorly controlled DMII, and CKD stage IIIa who presented acutely with chest pain. Her ECG showed ST-segment depressions and T-wave inversion in leads II, III, and aVF. Initial troponin I was positive at 9 mg/mL and formal transthoracic echocardiogram showed an EF of 35%-40%, with inferior wall hypokinesis.
Emergent CAG	CAG revealed a poorly visualized RCA with minimal anterograde flow. A Sion blue guidewire was used to cross into the rPDA branch. During predilation with a 1.5-mm balloon, a Ellis type III perforation occurred with contrast extravasation into the pericardial space.
Initial complication management	Balloon tamponade was applied to the perforation site to obtain control at the site of the bleed. A TTE was than performed which showed echocardiographic signs of tamponade including RV compression; 220 cm^3^ of hemorrhagic fluid was drained using a pigtail catheter.
Lesion management	The risks of reversing the heparin and causing an acute thrombosis of the thrombotic RCA was weighed against the possibility of continued bleeding if not reversed. As there were signs of ischemia including regional wall motion changes on formal echocardiogram, it was likely that reversing the heparin and allowing the RCA to infarct and cause hemodynamic instability. With a balloon in place tamponading at the rPDA site, a second JR4 catheter was used to engage the RCA. Once engaged a microcatheter was advanced into the rPL branch. Once wire access was obtained the lesion was ballooned, underwent penumbra, and stenting of the main RCA lesion.
Immediate outcome	After the intervention, her chest pain resolved and follow up TTE showed no reaccumulation of the pericardial effusion. During the same admission a staged intervention to the mid LAD was performed and repeat echo showed a recovered EF of 60%.
Late complication management	At the 3-month follow-up, she was noted to have intermittent chest pain and coronary angiography showed a 14 × 16 mm pseudoaneurysm at the site of perforation. She underwent coil embolization of the pseudoaneurysm while maintaining patency of the RCA. She tolerated the procedure well and has had no further presentation to hospital with angina.
Long-term follow-up	At the 6-month follow-up, the patient remained asymptomatic. Repeat TTE showed LVEF recovery to 60%. All stents were confirmed patent on angiography.

CAG = coronary angiography; CKD = chronic kidney disease; DMII = diabetes mellites type II; ECG = electrocardiogram; LAD = left anterior descending artery; LVEF = left ventricular ejection fraction; RCA = right coronary artery; rPDA = right posterior descending artery; rPL = right posterolateral artery; TTE = transthoracic echocardiogram.

## History of Presentation

A 79-year-old woman presented with acute-onset substernal chest pain, starting 2 hours prior. The pain was severe, constant, nonradiating, and unresponsive to sublingual nitroglycerin, raising concern for ischemia. The patient reported recent emotional distress, but denied other symptoms.

**Take-Home Messages**

• In managing coronary perforations during ACS, the ping-pong technique offers a viable strategy to maintain vessel patency without reversing anticoagulation, while early use of intracoronary imaging can help guide intervention strategy and potentially prevent complications.
• Long-term surveillance is essential as delayed complications like pseudoaneurysms may develop even after successful initial management.

On arrival, she appeared distressed and diaphoretic. Vital signs were stable (blood pressure, 110/60 mm Hg; heart rate, 95 beats/min; respiratory rate, 16; SpO₂, 98%). Physical examination showed no peripheral edema or heart failure signs, but persistent pain indicated ongoing ischemia. Despite taking aspirin en route, her symptoms warranted urgent evaluation for acute coronary syndrome (ACS), given her age and comorbidities, which increased the risk of adverse cardiovascular outcomes.

## Past Medical History

The patient had poorly controlled diabetes, hypertension, and stage 3 chronic kidney disease (baseline creatinine, 1.1 mg/dL) without recent decline. The patient communicated noncompliance with taking prescribed medications. She had a 30 pack-year smoking history, although she quit 5 years ago. Notably, she had no prior myocardial infarction, coronary interventions, or coronary artery bypass grafting.

## Differential Diagnosis

Given her persistent chest pain and high-risk profile, ACS was the primary diagnosis. However, other possibilities included coronary vasospasm, Takotsubo cardiomyopathy (linked to emotional stress), myocarditis (although less likely without elevated inflammatory markers), and aortic dissection (ruled out clinically). Her comorbidities suggested a multifactorial presentation, warranting an immediate workup to confirm the cause.

## Investigations

**Figure 1:**
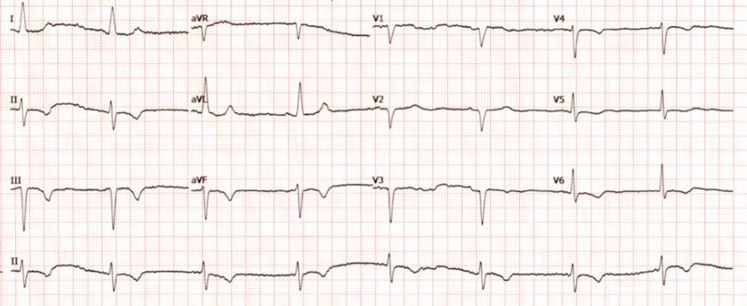
Presenting ECG With NSTEMI ST-segment depressions with T-wave inversions in the inferior leads. ECG = electrocardiography; NSTEMI = non-ST-segment elevation myocardial infarction.

An initial electrocardiogram showed ST-segment depression and T-wave inversions in the inferior leads (II, III, and aVF) with reciprocal changes in the lateral leads, suggesting right coronary artery (RCA) involvement (Figure 1). Troponin I was elevated at 9 ng/mL, indicating significant myocardial injury. Hemoglobin, electrolytes, and creatinine were normal, with creatinine at her baseline of 1.1 mg/dL. Pro-BNP was mildly elevated at 700 pg/mL, indicating ventricular strain. Transthoracic echocardiogram showed a left ventricular ejection fraction (LVEF) of 35% to 40% with inferior wall hypokinesis, confirming regional myocardial dysfunction and guiding procedural planning.

## Management and Procedural Details

**Figure 2:**
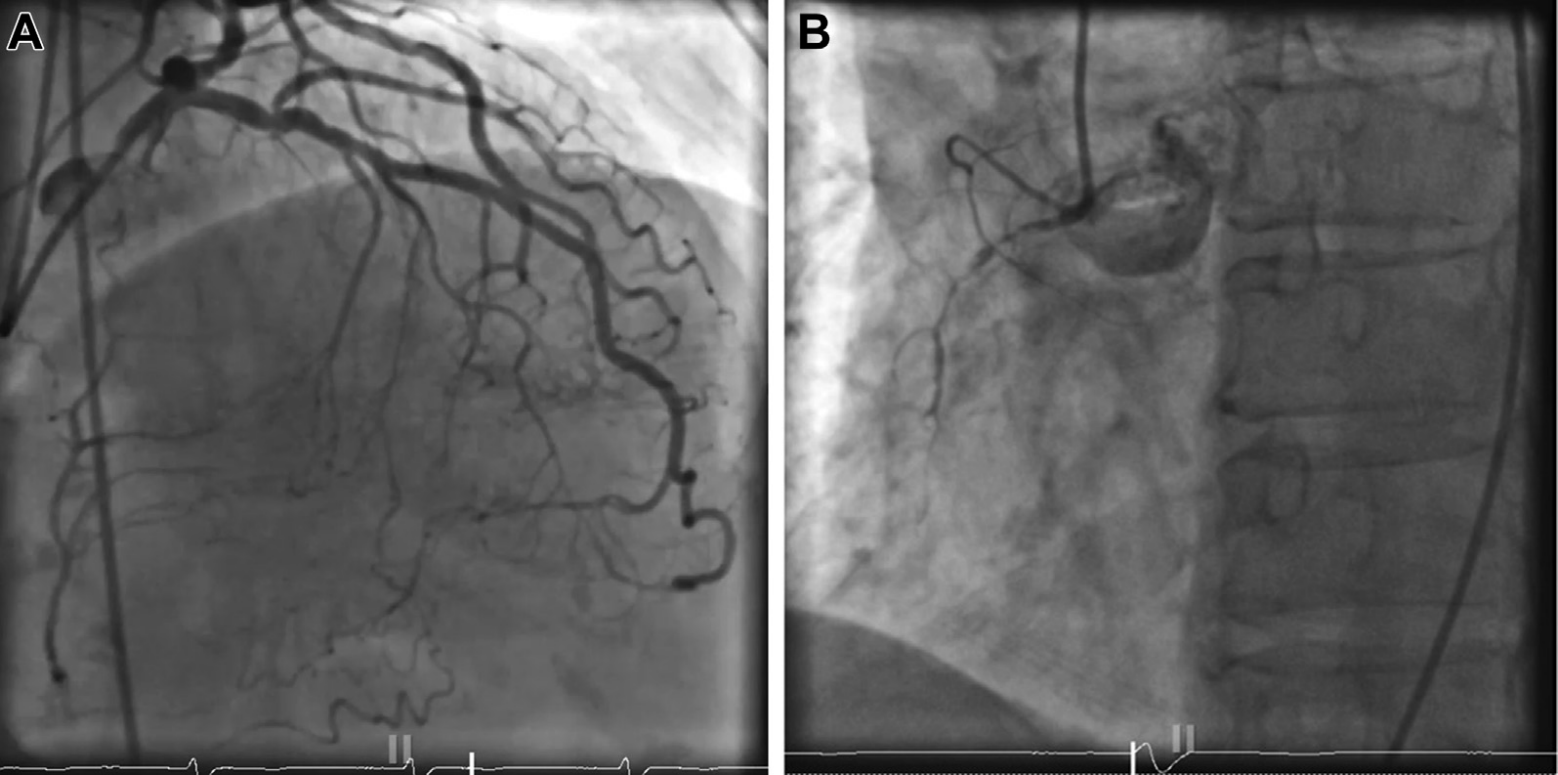
Angiogram (A) Left coronary angiogram. Mid left anterior descending disease with left to right collateral filling. (B) Right coronary angiogram. Occluded right coronary artery responsible for non-ST-segment elevation myocardial infarction.

The patient was promptly taken to the cardiac catheterization lab for coronary angiography. Initial imaging showed a poorly visualized RCA with minimal antegrade flow, raising suspicion for either diffuse thrombotic occlusion or a small-caliber vessel dependent on collateral flow from the left coronary system (Figures 2A and 2B).

**Figure 3:**
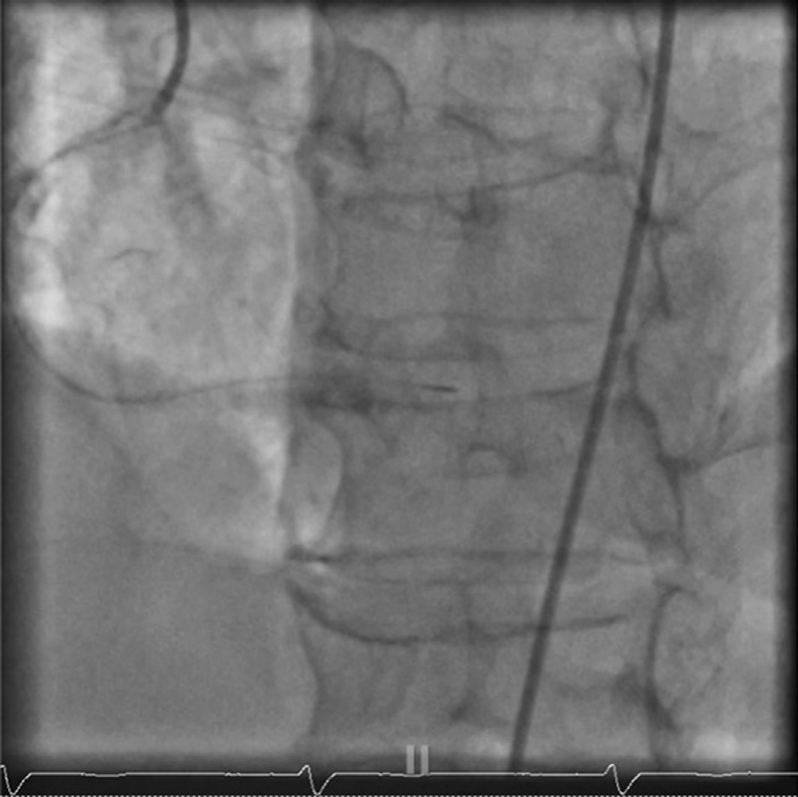
Native RCA Intervention Microcatheter advanced to distal RCA before predilation of RCA. RCA = right coronary artery.

**Figure 4:**
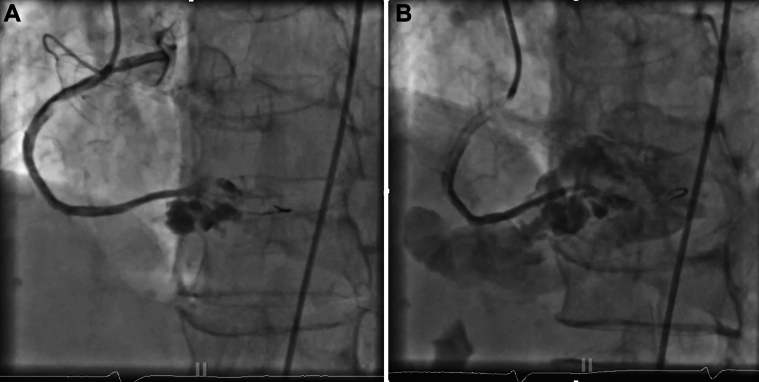
Type III Ellis Perforation (A) A type III Ellis perforation of distal RCA to rPDA after predilation of RCA with a 1.5-mm balloon. (B) A type III Ellis perforation of distal RCA to rPDA with contrast extravasation into the pericardial space. rPDA = right posterior descending artery; other abbreviation as in Figure 3.

Using a JR4 guide catheter, the team first attempted to cross the RCA with a Caravel microcatheter, but the device could not advance through the lesion. A switch was made to a Sion Blue guidewire, which successfully crossed what was identified as the right posterior descending artery (rPDA) branch (Figure 3). To improve visualization and establish patency, the vessel was predilated with a 1.5-mm balloon (Video 1). However, this maneuver resulted in a catastrophic Ellis type III perforation, with contrast extravasation into the pericardial space—a life-threatening complication (Figures 4A and 4B, Video 2).

**Figure 5:**
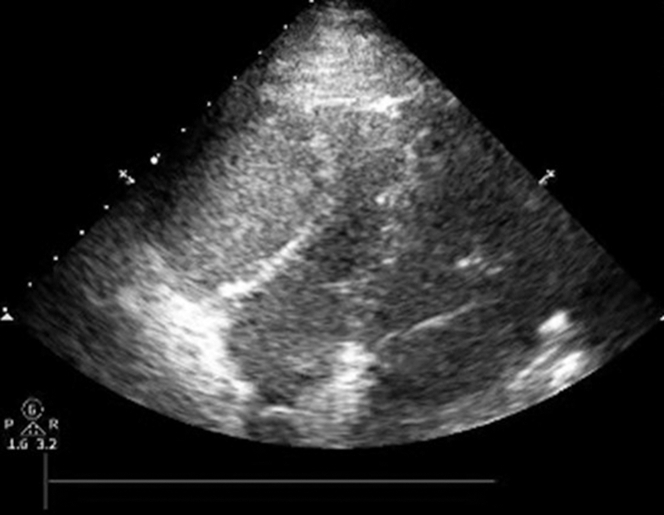
TTE Demonstrating Right Ventricular Compression TTE showing right ventricular compression concerning for tamponade. Subsequently, immediate epicardial drain was placed. TTE = transthoracic echocardiogram.

To manage the perforation, balloon tamponade was applied immediately to the perforation site to halt further bleeding. Transthoracic echocardiogram revealed right ventricular compression concerning for tamponade (Figure 5). Pericardiocentesis was performed using a pigtail catheter, and 220 cm^3^ of hemorrhagic fluid was drained from the pericardial space, stabilizing the patient’s hemodynamics.

This situation posed a critical dilemma: reversing heparin could reduce the bleeding risk, but it would also risk acute thrombosis of the RCA—an already thrombotic vessel. Collateral flow was present, but was weak and unreliable, and the patient demonstrated clear signs of ischemia, including elevated troponin, inferior ST-segment changes, and regional wall motion abnormalities on echocardiography. Allowing the RCA to infarct by sacrificing the vessel through permanent tamponade would have been a high-risk decision, likely leading to infarction and further hemodynamic instability.

**Figure 6:**
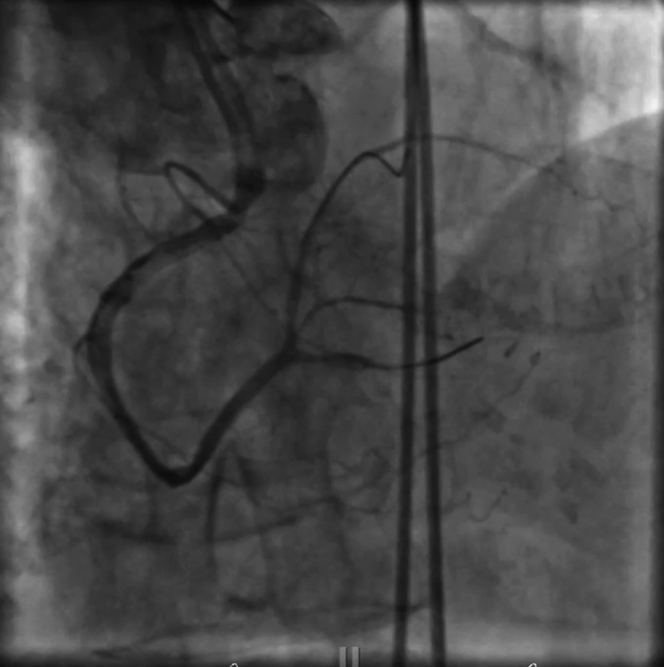
Ping-Pong Technique Right posterolateral artery wired and ballooned with good antegrade flow, while tamponade of right posterior descending artery with balloon was performed.

Given these competing risks, we attempted to maintain patency by creating outflow into the large right posterolateral artery (rPL) branch. We used the ping-pong technique, using a second JR4 guide catheter to reengage the RCA while keeping the tamponading balloon in place at the rPDA perforation site (Video 3). We successfully advanced a microcatheter into the rPL branch, establishing wire access (Figure 6).

While maintaining tamponade at the perforation, we performed balloon angioplasty along the entire RCA into the rPL, following thrombus aspiration with the Penumbra system. This maneuver restored antegrade flow throughout the RCA. We gradually deflated the tamponading balloon in the rPDA branch, confirming that no further bleeding occurred.

**Figure 7:**
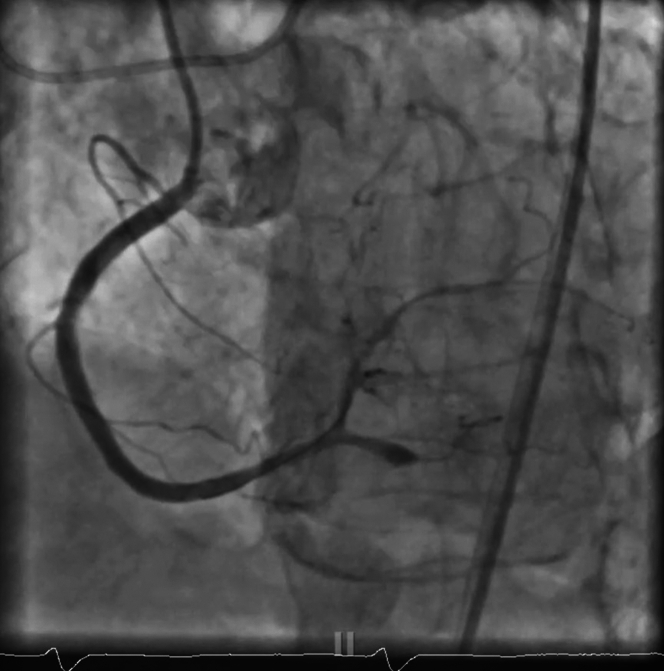
Successful Revascularization of RCA, rPL, and rPDA Concurrent successful revascularization with resolution of bleeding with balloon tamponade. TIMI III flow was restored to the RCA and rPL/rPDA accessory branch. rPDA = right posterior descending artery; rPL = right posterolateral artery; other abbreviation as in Figure 3.

A subsequent left coronary injection revealed the presence of an accessory rPDA branch, distinct from the main rPDA. After wiring both the rPDA and rPL, we performed balloon angioplasty to enhance outflow. The RCA was stented subsequently with intravascular ultrasound (IVUS) guidance to define the characteristics of the lesion, which showed thrombus along with superficial calcification <180° quadrant. We used a noncompliant balloon to predilate and stented with a 3.0-mm drug-eluting stent with excellent expansion. Post-stent IVUS examination ensured that there was no malapposition or residual thrombus (Figure 7, Video 4).

After the intervention, the patient’s chest pain resolved. A follow-up echocardiogram revealed a small pericardial effusion with mixed fibrinous material.

A staged intervention to the mid left anterior descending artery was performed during the same admission. At the 1-month follow-up, her LVEF had fully recovered to 60%, with all stents patent and no signs of recurrent ischemia.

**Figure 8:**
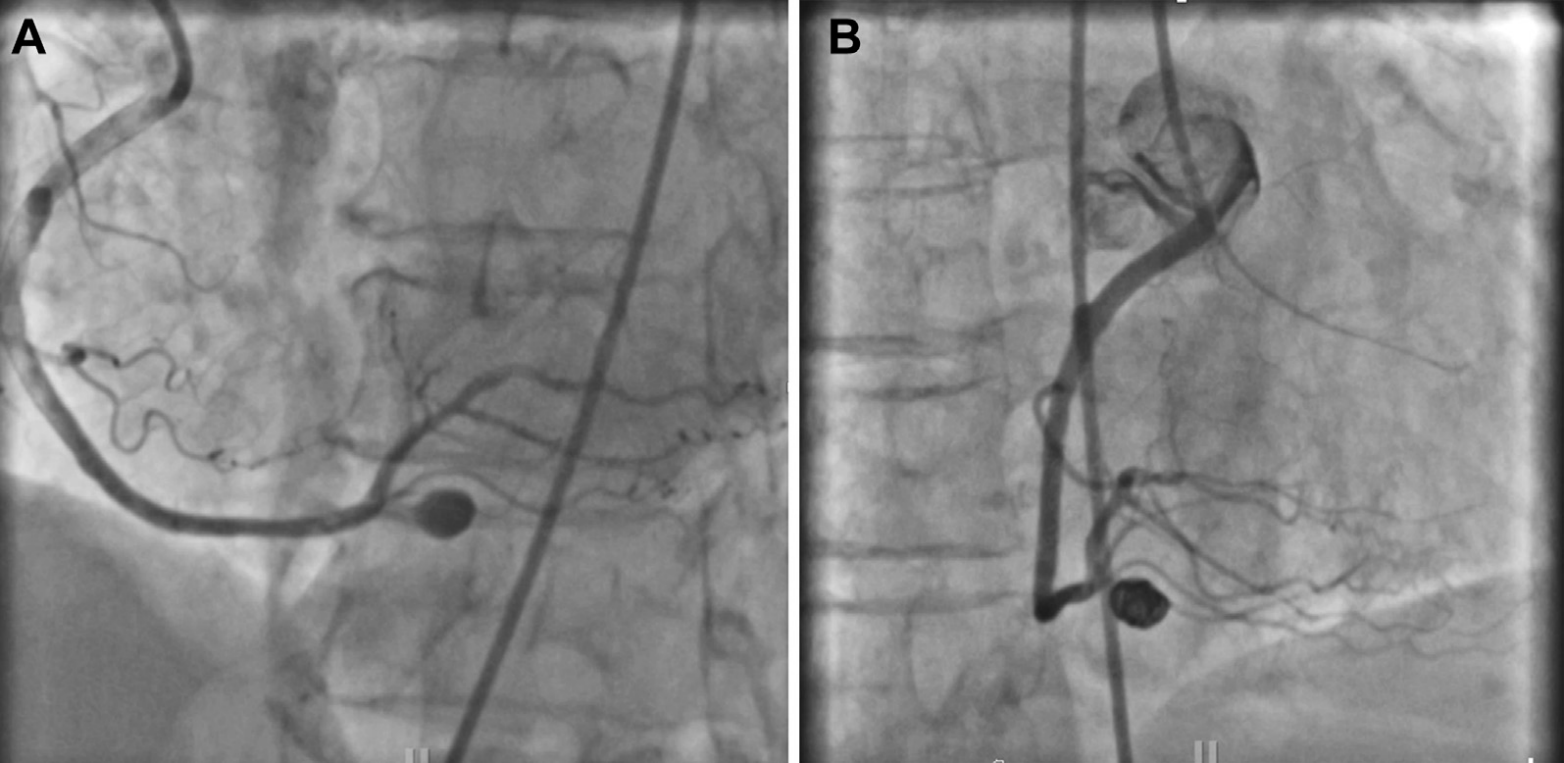
Follow-Up Imaging (A) Three-month follow-up pseudoaneurysm. Follow-up angiogram revealing rPDA Pseudoaneurysm on 3-month follow-up. (B). Coiling with successful isolation of the pseudoaneurysm. Successful exclusion of the pseudoaneurysm while preserving RCA patency including dual rPDAs. Abbreviations as in Figures 3 and 7.

At her 3-month follow-up, she reported intermittent chest discomfort, prompting repeat angiography. This imaging revealed a 14 × 16 mm pseudoaneurysm at the site of the previous perforation (Figure 8A). After discussing the treatment options—including surgical repair, covered stenting, and coil embolization—the patient elected coil embolization. This intervention successfully excluded the pseudoaneurysm while preserving RCA patency including the dual rPDAs (Figure 8B).

## Discussion

### Perforation preparedness: Anticipating and preparing for complications

In the lab, thorough preparation is essential to manage complications like pericardial tamponade, especially during high-risk coronary interventions. This begins with careful risk assessment, identifying high-risk features such as type B/C lesions, chronic total occlusions, calcified vessels, tortuous anatomy, and patient factors like advanced age or female gender.^1^ Proper sizing of equipment (maintaining balloon-to-artery ratios of ≤1.1) and careful use of high-risk devices, like atherectomy tools, are essential. Emergency supplies—pericardiocentesis kits, covered stents, and various balloon sizes—should be readily accessible. Medication preparedness is also crucial, with protamine available for heparin reversal and platelets on hand for GP IIb/IIIa inhibitor use. During procedures, careful guidewire positioning, frequent angiographic checks, and gentle handling in high-risk areas are recommended. Ensuring the team is briefed, surgical backup is available, and clear roles are assigned allows for a rapid, coordinated response if tamponade were to occur.^1^^,^^2^

### Perforation management: Challenges and options

This case highlights several important decision-making challenges in interventional cardiology, particularly the complexities involved in managing a type III coronary perforation during percutaneous coronary intervention (PCI). An Ellis type III perforation is classified as a severe perforation with free contrast extravasation, posing a significant risk of cardiac tamponade and hemorrhagic shock if not managed promptly.^1^^,^^3^

Coronary perforations remain rare but severe complications, with reported incidences ranging between 0.1% and 0.6% in PCI cases.^4^ Their management requires timely intervention and often the use of advanced techniques to mitigate bleeding without compromising vessel patency.

The dilemma of reversing anticoagulation becomes acute in such scenarios. Although reversing anticoagulation could mitigate bleeding risks, it increases the chance of stent thrombosis, myocardial infarction, or vessel occlusion significantly, especially during ACS. In the context of ACS, timely revascularization is essential to reduce infarct size and improve outcomes. Therefore, maintaining anticoagulation despite the bleeding risk is often the preferred approach.^1^^,^^3^

### The ping-pong technique: A dual-catheter approach

The ping-pong technique, which involves using 2 guide catheters—one to maintain tamponade and the other to continue intervention—plays a crucial role in managing complications during PCI. This technique is particularly valuable in scenarios such as coronary perforations, where maintaining hemostasis while continuing the intervention is essential.

The ping-pong technique is particularly valuable in other high-risk patients, such as those with CTOs or during complex interventions like transcatheter aortic valve replacement, because it allows for simultaneous bleeding control and revascularization.^5^ This dual-catheter approach mitigates the need to interrupt the procedure or reverse anticoagulation, which can lead to thrombotic events. For instance, operators may need to halt the procedure or reverse anticoagulation to control bleeding, which can lead to thrombotic events. The Society of Interventional Radiology, in their guidelines, emphasizes the importance of balancing anticoagulation to minimize thromboembolic risks while managing bleeding complications.^6^

The technique has been successfully used in treating perforations of grafts, such as the left internal mammary artery, by using a covered stent through a dual catheter approach, further enhancing procedural safety.^7^ The dual catheter method allows for the simultaneous use of a balloon to control bleeding and the delivery of a covered stent, which is crucial in managing severe perforations.^8^

In most cases, the ping-pong technique is used to manage active bleeding by maintaining tamponade at the perforation site, buying time for the deployment of covered stents or other definitive interventions. However, our case stands out for its unique approach—instead of solely focusing on halting the bleed, we aimed to preserve vessel patency.

Here, the ping-pong technique was not just a tool for bleeding control but a strategy to maintain outflow through the rPL branch. By tamponading the bleeding rPDA branch with 1 catheter while simultaneously wiring and ballooning the rPL branch with the second catheter, we ensured continuous antegrade flow. This allowed us to prevent vessel sacrifice and avoid infarction, while managing the perforation.

Our approach reflects a nuanced balance between hemostasis and revascularization, using the ping-pong technique not to merely “buy time” but to safeguard critical vessel patency.

### Intracoronary imaging to differentiate between thrombus and calcified disease

Before beginning any coronary intervention, it is crucial to determine the specific type of disease being treated. Chronic calcified disease and fresh thrombotic lesions require fundamentally different approaches. Severe calcific disease demands complex interventions involving multiple modalities, such as atherectomy, laser lithotripsy, and in some cases mechanical support. In contrast, thrombotic lesions are typically manageable with guide wire placement and thrombectomy when indicated.^9^

Intracoronary imaging is essential for properly assessing all lesions, regardless of their type. Whether using optical coherence tomography or IVUS examination, this imaging is critical for both acute thrombotic lesions and chronic calcifications. Even in acute cases, intracoronary imaging helps to identify hidden complexities, such as deep calcium deposits concealed behind large necrotic cores, which could impact the intervention strategy significantly.^10^

### Pseudoaneurysm management: Challenges and options

Pseudoaneurysms are a recognized complication after PCI, particularly after balloon tamponade or perforation. These aneurysms result from incomplete vessel wall rupture, with blood accumulating in a sac contained by surrounding tissue.^11^^,^^12^

Several treatment options exist for managing pseudoaneurysms. Coil embolization is a minimally invasive approach that isolates the pseudoaneurysm from circulation; in our case, Penumbra coils ensured stability without compromising flow.^11^ Covered stents can be used for aneurysms in large and stentable segments, but carry risks of thrombosis and side branch occlusion.^12^ Surgical repair is typically reserved for large, complex aneurysms when endovascular methods are insufficient.

## Outcome and Follow-up

At the 6-month follow-up, the patient remained asymptomatic with normal exercise tolerance. Repeat echocardiography showed LVEF recovery to 60%, and all stents were confirmed patent on angiography.

## Conclusions

Rapid decision-making and advanced techniques are crucial in managing life-threatening complications during PCI, as demonstrated by this case. The ping-pong technique proved effective in balancing hemostasis and vessel patency without reversing anticoagulation. Long-term follow-up remains essential, as evidenced by the successful management of the subsequent pseudoaneurysm.

## Funding Support and Author Disclosures

The authors have reported that they have no relationships relevant to the contents of this paper to disclose.

## Appendix

Video 1 Angioplasty Prior to Perforation The vessel was predilated with a 1.5 mm balloon which unfortunately resulted in perforation.

Video 2 Ellis Type III Perforation Predilation resulted in a catastrophic Ellis type III perforation, with contrast extravasation into the pericardial space.

Video 3 Ping-Pong Technique The ping-pong technique was used to tamponade the bleeding rPDA branch with one catheter while simultaneously wiring and ballooning the rPL branch with the second catheter.

Video 4 Resolution of the RCA Perforation The final result after the ping-pong technique was restoration of TIMI III flow to the RCA and rPL/rPDA accessory branch.
